# Exploring medically-related Canadian summer student research programs: a National Cross-sectional Survey Study

**DOI:** 10.1186/s12909-019-1577-z

**Published:** 2019-05-14

**Authors:** Sagar Patel, Catharine M. Walsh, Jacob A. Udell

**Affiliations:** 1 Faculty of Medicine, University of Toronto, King’s College Circle, Toronto, Ontario M5S 1A8 Canada; 2 Division of Gastroenterology, Hepatology and Nutrition, The Research and Learning Institutes, Department of Paediatrics, Hospital for Sick Children, Toronto, Ontario Canada; 3 Cardiovascular Division, Department of Medicine and Women’s College Research Institute, Women’s College Hospital, Toronto, Ontario Canada

**Keywords:** Medical education, Medical student, Medical careers, Summer student research programs, Clinician-scientists

## Abstract

**Background:**

Summer student research programs (SSRPs) serve to generate student interest in research and a clinician-scientist career path. This study sought to understand the composition of existing medically-related Canadian SSRPs, describe the current selection, education and evaluation practices and highlight opportunities for improvement.

**Methods:**

A cross-sectional survey study among English-language-based medically-related Canadian SSRPs for undergraduate and medical students was conducted. Programs were systematically identified through academic and/or institutional websites. The survey, administered between June–August 2016, collected information on program demographics, competition, selection, student experience, and program self-evaluation.

**Results:**

Forty-six of 91 (50.5%) identified programs responded. These SSRPs collectively offered 1842 positions with a mean 3.76 applicants per placement. Most programs (78.3%, *n* = 36/46) required students to independently secure a research supervisor. A formal curriculum existed among 61.4% (*n* = 27/44) of programs. Few programs (5.9%, *n* = 2/34) offered an integrated clinical observership. Regarding evaluation, 11.4% (*n* = 5/44) of programs tracked subsequent research productivity and 27.5% (*n* = 11/40) conducted long-term impact assessments.

**Conclusions:**

Canadian SSRPs are highly competitive with the responsibility of selection primarily with the individual research supervisor rather than a centralized committee. Most programs offered students opportunities to develop both research and communication skills. Presently, the majority of programs do not have a sufficient evaluation component. These findings indicate that SSRPs may benefit from refinement of selection processes and more robust evaluation of their utility. To address this challenge, the authors describe a logic model that provides a set of core outcomes which can be applied as a framework to guide program evaluation of SSRPs.

**Electronic supplementary material:**

The online version of this article (10.1186/s12909-019-1577-z) contains supplementary material, which is available to authorized users.

## Background

Clinician-scientists are uniquely qualified to integrate perspectives from their clinical experiences with scientific inquiry to generate new knowledge about health and disease through research and translate research findings into medical practice. However, despite the recognized value of integrating scientific discovery and clinical care, the decline in relative numbers of new clinician-scientists is well documented [1–8]. A number of recent reports have highlighted strategies to address this phenomenon, including the critical need to integrate recruitment efforts and research training across various stages of education [8–11]. A study by Silberman et al. [12] also indicated that most medical trainees decide whether to pursue a scientific career before entering residency training. Hence, opportunities to enhance involvement in medically-related research during earlier educational stages (i.e. undergraduate and medical school) provide a valuable opportunity to increase the numbers of well-trained clinician-scientists. Medically-related summer student research programs (SSRPs) targeting undergraduate and medical students represent one such avenue to increase students’ interest in a clinician-scientists career path.

Previous research has shown that SSRPs stimulate or strengthen students’ interest and participation in research activities, encourage integration of research into career choices, and aid in research-related skills development [9–25]. Many of the individual programs outlined in the literature have served to develop future clinician-scientists and increase researcher diversity by providing underrepresented students opportunities to become involved with research [15, 21, 23, 26–28]. To date, however, research regarding SSRPs has largely utilized self-reported data and described individual program successes within the United States (US) [15, 16, 19, 29, 30]. To our knowledge, there has been no previous comprehensive comparison of SSRPs across disciplines. Additionally, there remains a knowledge gap regarding the types of educational opportunities offered by such programs, the level of competition, and how programs measure impact and fulfillment of their implicit and explicit goals. This study, therefore, sought to understand the composition of existing medically-related Canadian SSRPs, describe the current selection, education and evaluation practices and highlight opportunities for improvement.

## Methods

This cross-sectional survey-based study took place from June to August 2016. Ethical approval was obtained from the Women’s College Hospital Research Ethics Board and verbal consent to participate was received from all participants.

### Program identification

Eligible summer student research programs were systematically identified using search engines, online databases and medical school websites. First, Canadian SSRPs were identified by making a list of all 17 Canadian medical schools and their affiliated partners, including academic hospitals, universities, laboratories and institutions. Second, to account for national programs, Canadian healthcare organizations that offered summer research studentships were identified by exploring medical school webpages. Using this comprehensive list, an online search was conducted to identify eligible programs. SSRPs that offered research placements within Canada, were based in the English language and independent of school credit, and specific to the summer months (May to August) were included. SSRPs open to students from non-medical disciplines, such as Masters, PhD, pharmacy and dentistry, were excluded. Programs were also excluded if they were unable to provide the research team with contact information for a designated research coordinator and/or director that could accurately answer the study questionnaire.

### Data collection

The authors developed a survey instrument based on a literature review, knowledge of current research training programs, discussions with experts in the field, and their own research training experience. Two investigators (JU and SP) reviewed the questionnaire to provide feedback on clarity and completion time. Additionally, the instrument was pre-tested on a sample of included respondents (*n* = 5) to ensure ease of use and relevance. Feedback was incorporated into the final instrument. The instrument consists of 32 questions divided into 8 sections designed to obtain information regarding program characteristics (*n* = 6), competition level (*n* = 4), accessibility (*n* = 2), student selection (n = 2), funding (*n* = 2), student experience (*n* = 7), program self-evaluation (*n* = 8) and other (*n* = 1). The survey (Additional file 1: Appendix 1) was administered via telephone interview (91.3% [42/46]) or written questionnaire in cases where the SSRPs’ representative was unavailable for a phone interview (8.7% [4/46]). A call script was used to standardize survey administration. To maximize response rates, the survey design and distribution was based on Dillman’s tailored design method [31], including clear and easy-to-understand language, personalized communication, a short cover letter and distribution of up to two reminder emails. Additionally, the option of responding to a written questionnaire was provided to enhance participation.

### Statistical analysis

Descriptive statistics were used to summarize survey responses and summary statistics, as means and/or raw number and percent, are reported where appropriate. Differences between responders and non-responders with respect to geographic region and institution type were assessed using chi square. Where applicable, data were stratified and analyzed according to program size to facilitate comparisons across programs with similar resources and infrastructure: small (≤20 seats), medium (20–50 seats), large (≥50 seats) and pan-Canadian programs available to student’s independent of the province in which they live.

## Results

**Fig. 1:**
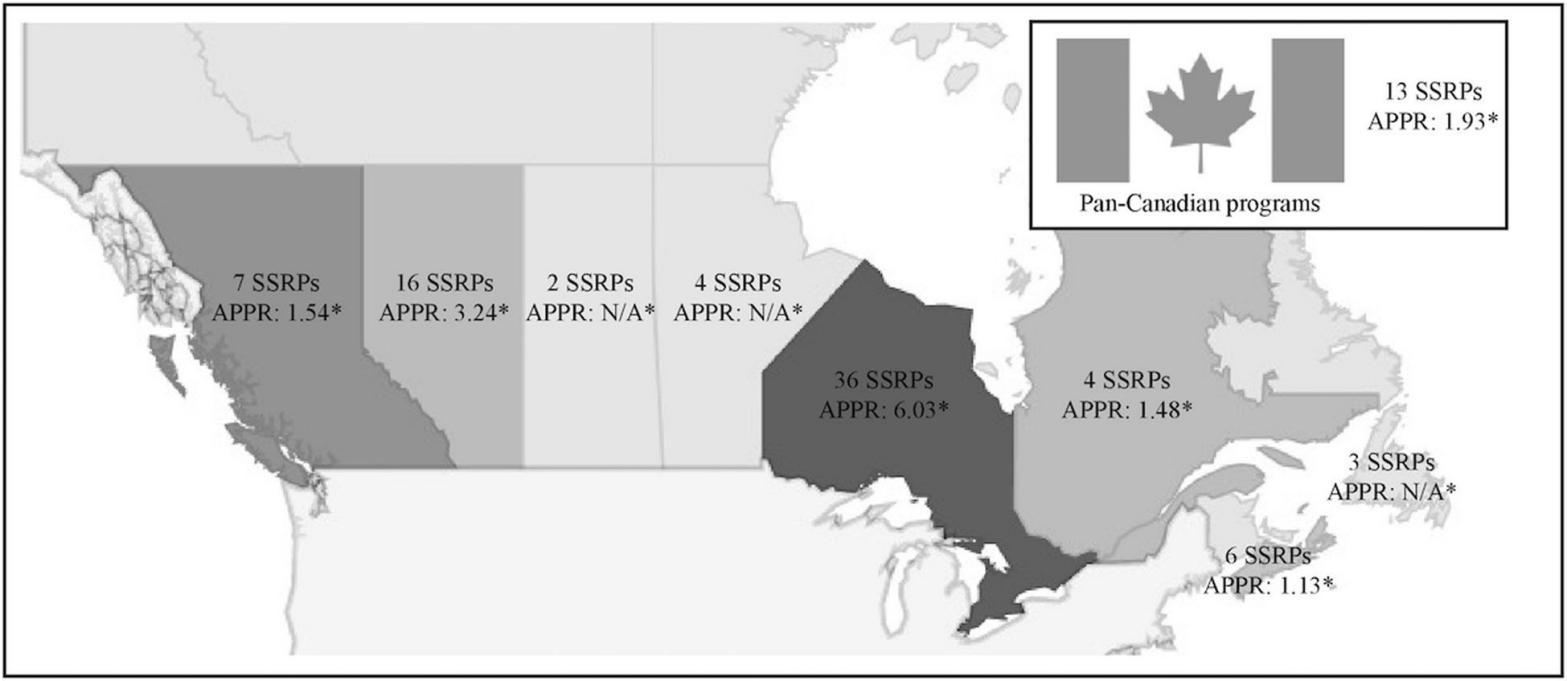
Heat map depicting competition level of SSRPs across Canadian provinces for 2016 as represented by the mean applicant to placement ratio (APPR). Darker grey colour represents higher completion level. APPR: mean applicant to placement ratio; SSRP: summer student research program. * Survey responses by provinces were as follows: British Columbia = 4, Alberta = 4, Saskatchewan = 0, Manitoba = 0, Ontario = 16, Quebec = 2, Nova Scotia/New Brunswick = 6, Newfoundland = 3, Pan-Canadian programs = 4

Ninety-one programs met inclusion criteria (See Additional file 1: Appendix 2 for complete list and Fig. 1 for national distribution of programs)**.** Four different types of institutions offered SSRPs, including Canadian Medical Schools and their affiliated research institutes and universities, and academic hospitals together with their associated research institutes. In many cases multiple different summer research programs were offered by the same institution. A comparison of types of institutions offering SSRPs across Canadian provinces is provided in Additional file 1: Appendix 3. Overall, 26 SSRPs were offered by Canadian medical schools, 12 by affiliated research institutes, 21 by universities and 19 by academic hospitals and their associated research institute. Forty-six of 91 (50.5%) identified programs participated in the study. Geographically, the distribution of response rates across Canada were 34.5% (10/29) for western Canada, 57.5% (23/40) for central Canada, 66.7% (6/9) for eastern Canada and 53.8% (7/13) for pan-Canadian programs. Amongst the types of institutions offering SSRPs, response rates were 53.8% (14/26) for Canadian medical schools, 42.9% (9/21) for affiliated universities, 41.7% (5/12) for affiliated research institutes, and 57.9% (11/19) for academic hospitals and their associated research institute. There were no differences between responders and non-responders with respect to geographic region or institution type (*p* > 0.05). Question completion rate was variable either due to missing data or the participants’ choice to not respond.

### General program characteristics

General program characteristics of Canadian SSRPs are presented in Table 1. Overall, most of the programs surveyed (78.3% [36/46]) had been in existence for greater than 5 years and offered both clinical (87.0% [40/46]) and basic (82.6% [38/46]) research opportunities. Greater than half of SSRPs (58.7% [27/46]) offered research positions at both the undergraduate and medical student level, and 23.9% of programs (11/46) were not restricted to students from a specific university. Less than half (34.8% [16/46]) of programs had a formal screening process for supervisor selection. Canadian SSRPs reported internal variations regarding the length of summer student employment, with 12-week (50.0% [23/46]) and 16-week (41.3% [19/46]) placements being most common. Funding came from a variety of contributions, including the host institution, principal investigator grants, private donations, government support and independent student funding. Disciplines of research offered by the programs are presented in Additional file 1: Appendix 4, with medicine being the most common and bioinformatics, biomedical engineering and physics being the least represented disciplines.

**Table 1:** General characteristics of Canadian summer student research programs for 2016

	Small (≤ 20 seats) *n* = 17	Medium (20–50 seats) *n* = 11	Large (≥ 50 seats) *n* = 11	Pan-Canadian *n* = 7	Total *n* = 46
Mean number of positions per program	11	28	111	18	40
Class of research offered					
Clinical	14 (82.4%)	9 (81.8%)	11 (100.0%)	6 (85.7%)	40 (87.0%)
Basic	13 (76.5%)	7 (63.6%)	11 (100.0%)	7 (100.0%)	38 (82.6%)
Field work	5 (29.4%)	3 (27.3%)	6 (54.5%)	4 (57.1%)	18 (39.1%)
Other^*^	2 (11.8%)	3 (27.3%)	1 (9.1%)	0 (0%)	6 (13.0%)
Educational requirements					
Undergraduate students	5 (29.4%)	6 (54.5%)	2 (18.2%)	0 (0%)	13 (28.3%)
Medical students	0 (0%)	1 (9.1%)	2 (18.2%)	3 (42.9%)	6 (13.0%)
Both	12 (70.6%)	4 (36.4%)	7 (63.6%)	4 (57.1%)	27 (58.7%)
Program restricted to specific universities					
Yes	2 (11.8%)	3 (27.3%)	6 (54.5%)	0 (0%)	11 (23.9%)
Mean age of program					
< 5 yrs.	2 (11.8%)	4 (36.4%)	2 (18.2%)	2 (28.6%)	10 (21.7%)
> 5 yrs.	15 (88.2%)	7 (63.6%)	9 (81.8%)	5 (71.4%)	36 (78.3%)
Duration of program					
8 weeks	7 (41.2%)	1 (9.1%)	4 (36.4%)	2 (28.6%)	14 (30.4%)
10 weeks	3 (17.6%)	2 (18.2%)	3 (27.3%)	2 (28.6%)	10 (21.7%)
12 weeks	12 (70.6%)	3 (27.3%)	7 (63.6%)	1 (14.3%)	23 (50.0%)
14 weeks	3 (17.6%)	5 (45.5%)	3 (27.3%)	1 (14.3%)	12 (26.1%)
16 weeks	8 (47.1%)	7 (63.6%)	4 (36.4%)	0 (0%)	19 (41.3%)
Did not specify	0 (0%)	0 (0%)	0 (0%)	1 (14.3%)	1 (2.2%)
Selection of supervisors					
Formal screening	7 (41.2%)	1 (9.1%)	4 (36.4%)	4 (57.1%)	16 (34.8%)
No formal screening	8 (47.1%)	9 (81.8%)	4 (36.4%)	1 (14.3%)	22 (47.8%)
Did not specify	1 (5.9%)	1 (9.1%)	3 (27.3%)	2 (28.6%)	7 (15.2%)
Funding^†^					
Host institution	8 (47.1%)	7 (63.6%)	6 (54.5%)	2 (28.6%)	23 (50.0%)
Principal investigator grant	8 (47.1%)	6 (54.5%)	7 (63.6%)	0 (0%)	21 (45.7%)
Private donations	6 (35.3%)	3 (27.3%)	5 (45.5%)	6 (85.7%)	20 (43.5%)
Government support	2 (11.8%)	2 (18.2%)	2 (18.2%)	0 (0%)	6 (13.0%)
Student secured funding	0 (0%)	1 (9.1%)	1 (9.1%)	0 (0%)	2 (4.3%)

*Other: outcomes research, basic research on humans, dry labs, animal research, computer lab or design lab; ^†^Options not mutually exclusive

### Competition, accessibility and student selection

The 46 programs collectively offered 1842 positions. Competitiveness of entry varied by program size and geographical location, as presented in Fig. 1. The competition level is reported as applicants per placement ratio (APPR). Small (94.1% [16 /17 responded]), medium (63.6% [7/11 responded]) and large programs (72.7% [8/11 responded]) reported a competition level of 4.27, 4.46 and 3.06 APPR, respectively. Pan-Canadian programs (4/7 responded [57.1%]) reported a competition level of 1.93 APPR. Amongst respondents (76.1% [35/46]), the mean APPR was 3.76:1. Half of the programs (50.0% [23/46]) reported on the proportion of female applicants and accepted female students, which were 53.7 and 53.0%, respectively. Less than one tenth of SSRPs (6.5% [3/46]) reported special consideration and/or reserved spots for underrepresented minorities during the interview and/or hiring process.

The majority of programs reported on their application requirements (93.5% [43/46]), with 41.9% of programs (18/43) requiring a minimum GPA cut-off and 58.1% (25/43) necessitating a reference letter. Less than half of SSRPs (44.2% [19/43]) requested a letter of interest detailing the applicant’s interest in a research-oriented career, a career in medicine (4.7% [2/43]) or both (16.3% [7/43]). Most programs (81.8% [36/44]) required students to independently find a principal investigator (PI) to supervise their research experience, while only 18.2% (8/44) had a centralized oversight committee to screen and assist students’ matches with PIs.

### Student experience

Table 2 outlines the learning opportunities available to students. Of the programs offering clinical research experience, chart-based research was most common (73.0% [27/37]), while few programs (29.7% [11/37]) offered opportunities to conduct research within the operating room setting. Very few (5.9% [2/34]) SSRPs that were based within an academic health centre combined student’s research experience with a mandatory integrated clinical observership. As a supplementary educational component, the majority (61.4% [27/44]) of programs held educational research rounds and/or teaching sessions. The most common forms of presentation experience required by SSRPs included internal oral (43.5% [20/46]) and poster (41.3% [19/46]) presentations.

**Table 2:** Learning opportunities available to summer students for 2016

If clinical research is available: what type of clinical exposure do student receive? (*n* = 37 respondents)	
No contact with patients or patient charts	18 (48.6%)
Patient charts	27 (73.0%)
Oral communication with patients	25 (67.6%)
Physical contact with patients	16 (43.2%)
Operative room exposure	11 (29.7%)
Not tracked	5 (13.5%)
If the program/institution is affiliated with an academic health care center, do students get an opportunity to shadow/observe clinicians? (*n* = 34)	
Required component	2 (5.9%)
Not a required component	5 (14.7%)
Upon the discretion of the supervisor	27 (79.4%)
Does the summer student research program incorporate research rounds and/or teaching sessions? (*n* = 44)	
Yes	27 (61.4%)
No	17 (38.6%)
If applicable: How often are research rounds and/or teaching sessions held for the students? (*n* = 24)	
Weekly	15 (62.5%)
Biweekly	5 (20.8%)
Monthly	1 (4.2%)
Other	1 (4.2%)
How are the students required to present their work at the end of the term? (*n* = 46)	
Internal oral presentation	20 (43.5%)
External oral presentation	5 (10.9%)
Internal poster presentation	19 (41.3%)
External poster presentation	6 (13.0%)
Abstract/manuscript submission	3 (6.5%)
Written report of experience/Summary of results for donors/PI	5 (10.9%)
No form of presentation required	9 (19.6%)

### Program self-evaluation

Table 3 illustrates the usage of program self-evaluation strategies, such as measurement of student’s research productivity through resultant publications, collection of student and PI feedback, and students’ future career trajectories. Very few (11.4% [5/44]) programs reported collecting resultant student publications centrally. Although many (65.2% [30/46]) programs elicited student feedback, a smaller number (32.6% [15/46]) reported collecting PI feedback. Additionally, most (87.5% [35/40]) SSRPs did not follow up with students after program completion.

**Table 3:** Canadian SSRP self-evaluation strategies

Tracking of resultant student publications (*n* = 44)	
Yes	5 (11.4%)
No	39 (88.6%)
Feedback collected (*n* = 46)	
Student Feedback	30 (65.2%)
PI Feedback	16 (34.8%)
Feedback not collected	15 (32.6%)
Does the program centrally track where students are in their training and/or career after completion of the summer research program? (*n* = 40)	
Centrally monitored	11 (27.5%)
1–3 years	5 (12.5%)
3–5 years	4 (10.0%)
> 5 years	4 (10.0%)
Not centrally monitored	35 (87.5%)

## Discussion

This national survey provides, to our knowledge, the first comprehensive comparison of medically-related SSRPs. Overall, competition for SSRP positions was strong across Canada. Ontario and Alberta had the highest rates of competition, while pan-Canadian programs had relatively low competition rates. We identified that the most common hiring approach required students to independently find support from a research supervisor before applying to a program. Regarding supplementary educational opportunities, the majority of programs offered educational seminars and opportunities for students to enhance their oral presentation skills. Very few programs situated within an academic healthcare center had an integrated clinical observership as a mandatory component and the majority of students performing clinical research did not have direct patient contact. Finally, most programs did not track resultant student publications and few programs followed students after completion of their summer experience. Most performance review effort and post-program student follow-up was left to the discretion of the individual PI.

The growing shortage of clinician-scientists make early intervention and exposure to research a priority [9]. No previous study has comprehensively described the characteristics of existing SSRPs on a national scale. Agarwal et al. [13] sought to review SSRPs, but their study was limited in that it focused solely on national radiation oncology research programs in the US and only examined opportunities at the medical student level. The study surveyed five national programs and reported on a few general characteristics, including program length and the class of research offered [13]. Most of their surveyed SSRPs were 6–10 weeks long which, on average, was shorter than our findings. Solomon et al. [25], who examined SSRPs from two medical schools in the US, found that a positive summer research experience was associated with increased interest in a research career. Additionally, long-term follow-up data (minimum 8 years) revealed that alumni were more likely to pursue an academic and/or research career, as compared with their classmates [25]. Our study builds on previous research as it provides a more comprehensive comparison of programs on a national scale.

Our results highlight several important areas for potential improvement in regard to student selection, administration, curriculum and program evaluation. First, trends observed suggest that the selection of SSRP placements fell primarily to the independent research supervisors, as opposed to a central committee. A non-centralized student selection process may reduce student diversity through biases posed against specific cohorts of students and/or interpersonal connections; an area of particular concern as women and individuals from underrepresented groups represent a very small percentage of the clinician–scientist workforce [32, 33]. An oversight committee may help to mitigate selection bias, such as the one developed by the Michigan State University research education program which was associated with a diverse student body [18]. However, an oversight committee has potential limitations of increased cost and time to administer the program.

Second, we found that many programs offered opportunities for student growth through educational seminars and student presentations. A number of published program evaluations highlight the effectiveness of these supplementary opportunities for the development of technical research skills and non-technical skills, such as effective written communication and public speaking [17, 19, 27]. Further supplementary opportunities may involve offering clinical exposure as part of the summer research experience. Among programs affiliated with an academic health care center, very few offered a formal integrated clinical observership as part of their curriculum. The benefits and implication of incorporating clinical observerships has not been formally evaluated. However, recommendations stemming from studies on clinician scientist-training programs have underscored the need for clinical-research integration [10, 34]. These recommendations work under the assumption that concurrent clinical and research exposure enables students to appreciate the importance of basic research, research application and the symbiotic relationship between research and practicing medicine [10, 35, 36]. Such benefits have been reported by a few programs, including the Rural Summer Studentship Program at The University of Western Ontario [37]. A retrospective analysis of the program showed that clinical learning combined with a research component increased students’ knowledge about rural medicine and stimulated students’ interest in rural medicine both clinically and academically [37]. Similar evidence has been reported by individual programs in the US that utilized clinical observerships as a medium for students to better understand the relationship between research and practicing medicine [27, 38].

Third, our findings suggest that improved self-evaluation strategies are required to help programs monitor their impact on students and the scientific community. Self-evaluation could also help track the achievement of a program’s implicit and explicit goals [39]. These goals might be as fundamental as providing students an opportunity to practice research skills to as specific as generating interest in becoming a palliative care researcher [27, 37]. Successful implementation of short-term impact surveys and longitudinal tracking surveys have been modeled by National Cancer Institute funded short-term cancer research training programs [40]. Our results suggest that most programs in Canada do not track resultant student publications nor do they follow-up with students after program completion. Objective quantitative metrics of program success, such as publication rate, could help support SSRPs if they are applying for funding and/or support to improve or expand their program [41]. Such metrics could also be used to evaluate the effectiveness of curricular changes. Student follow-up may also serve as a form of self-evaluation to identify short- and long-term impacts of a SSRP on students’ research abilities, professional identify formation and future career trajectories. University alumni records have been shown to facilitate longer term tracking of program graduates’ contact information and automated literature searches facilitate identification of post-training publications [42, 43]. Social networking tools, such as Facebook and LinkedIn, are reported to be ineffective in longitudinal tracking of program alumni career path and professional achievements [42]. Utilization of newer social networking tools, such as ResearchGate©, and unique digital identifiers for research contributors, such as Open Researcher and Contributor IDs (ORCID), have not been explored and may serve as a more effective platform to track longer term research productivity of previous program alumni.

The lack of self-evaluation strategies reported may be attributed to the complexity of SSRPs. For example, institutional context, quality of supervision, differences in individual research projects and format of learning experiences. For this reason, traditional methods of program evaluation may not be feasible and/or accurate in measuring program impact. To address this challenge, we described a logic model which provides a framework to guide program evaluation of SSRPs (Additional file 1: Appendix 5). A logic model is a systematic and visual way of presenting the relationships among the operational resources of a program (inputs), program activities, the immediate results of a program (outputs) and desired program accomplishments (outcomes) [44, 45]. Table 4 highlights both program-related outcomes and system-wide outcomes stemming from the model that can be targeted for evaluation [46]. Understanding that SSRPs, in general, have differences in objectives and mission statement we have designed the model with the mind-set of flexibility. Collection of self-evaluative data would serve as a resource to allow future students and investigators to quantify and accurately assess the value of an individual SSRPs and compare outcomes across programs. Additionally, evaluation data could be used to help better define characteristics of effective SSRP programs and, ultimately, to help optimize training and support for those individuals who are poised to develop into clinician-scientists.

**Table 4:** Program-related and system-wide logic model outcomes which can be applied as a framework to guide the evaluation of Summer Student Research Programs

Program-Related Outcomes (Proximal Outcomes) Short, intermediate and long-term benefits of program activities	
*For Students:* • Improved research knowledge and skills^†^ • Enhanced self-efficacy and identity as a researcher^†^ • Resume building for future jobs/professional school applications/residency^†^ • Managing commonly faced realities of research (e.g. tight research deadlines, limited resources, etc.) ^†^ • Improve writing and oral presentation ability^†^ • Improved knowledge of topics presented within curriculum^†^ • Strengthen professional network and support systems^†^ • Increase confidence and interest in conducting research^†^ • Earlier identification of medicine/surgical specialty interests (e.g., geriatrics, rural, radiology) ^†^ • Increased awareness of research application^†^ • Evidence of continued commitment to developing a research career^‡^ • Subsequent research initiated that includes networks developed during SSRP^‡^ • Experiential learning: broadening/reinforcing learning through completion of a subsequent research project^‡^ • Improve role transition into clinician-scientist^§^ • Foster and strengthen interest to pursue postgraduate research training/degree(s) ^§^ • Community of practice that supports research career development ^§^ • Community of SSRP graduates who promote research by giving back to their program ^¥^ *For Supervisors:* • Networking for mentors^†^ • Strengthening mentorship skills (e.g., providing feedback, setting expectations) ^†^ • Research team management skills^‡^ • Subsequent graduate student recruitment/supervision of SSRP students^‡§^ *For Program:* • Demonstrating recognition and value of research^‡^ • Forming a community of researchers (faculty and trainees) ^‡^ • Refinement of goal setting and evaluative strategies^‡^ • Developing future research leaders^§^	
System-Wide Impacts (Distal Outcomes)	
• Increase number of clinician-scientists^§^ • Increase researcher diversity^§^ • Increase knowledge translation^§^ • Program graduates who are editors of journals^§^	

^†^Short (≤1 year), ^‡^intermediate (1–3 year) and ^§^long-term (≥3 years) outcomes provided

### Limitations

This study had several limitations. First, there is no centralized directory of Canadian SSRPs, so it is possible that despite our efforts we may have missed some programs in our systematic search. Secondly, our overall response rate was 50.5%, with a lower response rate for western Canada (34.5%). Although typical for survey-based research [47], it leaves significant room for non-response bias and may serve to limit the generalizability of the results. We believe our sample is representative as the respondents were from diverse institutional types across Canada and there were no systematic differences between responders and non-responders with respect to geographic region or institution type. Thirdly, we limited our search to English-language-based programs, thus excluding French programs, based predominantly in Quebec. In an effort to develop a feasible survey we may not have captured the full breadth of available program data. Follow-up semi-structured interviews would have been a useful method to capture such data. It is also difficult to interpret the mixed reporting of summer programs for undergraduates and those for medical students. The two groups may have different motivations for participation; however, the need for evaluation of programs remains essential. The high competition level we described may be influenced by students applying to multiple SSRPs which our survey could not discern. Finally, due to administrative and logistical complexities of SSRPs it was sometimes difficult for program directors and/or coordinators to select survey responses that perfectly mirrored their unique processes and circumstances.

## Conclusions

Our national survey of Canadian SSRPs provides an up-to-date picture of current practices. Overall, our findings suggested that SSRPs were highly competitive. The student selection process was primarily the responsibility of individual research supervisors; a task which may be better suited for a centralized oversight committee to decrease the administrative burdens placed on PIs and reduce the potential for bias and favouritism during the student selection process. While most programs provided students with supplementary educational opportunities, clinical exposure was rare. Very few programs have a robust formal self-evaluation plan to monitor fulfillment of their implicit and explicit goals. Programs should be encouraged to implement quantitative and qualitative self-evaluative strategies, such as tracking resultant student publications and long-term impact assessments. The logic model provided affords a framework of core outcomes that can be utilized to guide program evaluation of SSRPs and prioritize outcomes evaluation.

## Additional file

Additional file 1: Supplemental Digital Content (DOC 347 kb)

## Abbreviations

APPR: Applicants per placement ratio
PI: Principal Investigator
SSRPs: Summer Student Research Programs
US: United States
