# Supplementary material for: Exploring medically-related Canadian summer student research programs: a National Cross-sectional Survey Study
Source: BMC Med Educ. 2019 May 14;19:140. doi: 10.1186/s12909-019-1577-z (PMC6515625; doi:10.1186/s12909-019-1577-z)
Supplement: Supplementary file 1 — Supplemental Digital Content (DOC 347 kb) [file 12909_2019_1577_MOESM1_ESM.doc]

**Supplemental Digital Content**

**Contents**

Appendix 1: Survey instrument …………………………………………………….…………..2-6

Appendix 2: Eligible Canadian summer student research programs (n = 91) …………......….7-11

Appendix 3: Comparison of institutional types offering summer student research programs across Canada in 2016 ……………………………………………………………………….….12

Appendix 4: Disciplines of research offered by Canadian SSRPs (n = 46) for 2016………........13

Appendix 5: Logic model providing a framework of core outcome sets to guide program evaluation of summer student research programs………………………………………………..14

**Appendix 1:** Survey instrument

**Section 1: Program Characteristics**

1. Educational requirements to be eligible for the summer studentship:

Only undergraduates

Only medical Students

Undergraduates & medical students

1. Is the summer student research program confined to certain universities or medical schools?

Yes

No

1. Duration of Summer Program:

8 weeks

10 weeks

12 weeks

14 weeks

16 weeks

1. How many years has this program been running for?

<1 year

1-3 years

3-5 years

5-10 years

> 10 years

1. How many scientists or mentors (approx.) are available to mentor or supervise prospective students? Is there a screening process for principal investigator (PI) selection?

PIs are selected with some form of qualification or screening procedure

1. What disciplines are involved with the research program?

Obstetrics or gynecology

Medicine  Family

Surgery

Psychiatry or psychology

Radiology

Public health or epidemiology

Pediatrics

Women’s health

Social work

Medical education

Health care system or quality control

Other health disciplines:

**Section 2: Competition level**

1. Total Number of positions available in 2016:

1. Number of applicants in 2016:

Institution has record but does wish to disclose

Information not tracked by the program

1. What proportion of applicants were female in the most recent application cycle?

Institution has record but does wish to disclose

Information not tracked by the program

1. Successful proportion of enrolled students that were female in the most recent application cycle:

Institution has record but does wish to disclose

Information not tracked by the program

**Section 3: Accessibility**

1. Has the program offered special consideration for the following groups?

Indigenous

Financial need

Disabled

Other

No special considerations offered

1. Does the program track the participation of students that fall under the category of disadvantaged, underrepresented or a minority group?

Indigenous

Financial Need

Disabled

Other

Not tracked

**Section 4: Student Selection**

1. Application requirements:

Minimum Grade Point Average (GPA)

Letter of reference(s)

Letter of interest to pursue a career in research

Letter of interest to pursue a career in a specific field (e.g. medical imaging, etc.)

1. Recruitment Procedure:

Student must securely obtain a suitable principal investigator before applying to the program.

Students apply to the program and are screened centrally. Successful applications are then circulated amongst principal investigators affiliated with the program, who then make the decision of interviewing them for a potential spot.

Other:

**Section 5: Funding**

1. How are the students compensated?

Stipend

Payroll

Honorarium

Other

1. How are the studentships financed?

Government supported program

Private donor contribution or donations or not for profit organization

Host institution

PI grant

Students are required to apply for their own funding

Other:

**Section 6: Student Experience**

1. Types of research positions available:

Wet Labs

Clinical Research

Field Work

Other

1. If clinical research is available: what type of clinical exposure do student receive?

No contact with patient or patient charts

Patient charts

Oral communication with patients

Physical contact with patients

Operative room exposure

Other:

1. If the program or institution is affiliated with an academic health care center, do students get an opportunity to shadow or observe clinicians?

Required component of the summer student research program

Not a required component, however program actively encourages mentors to allow students an opportunity to shadow or observe

Upon the discretion of the PI, program doesn’t encourage or discourage such opportunities.

N/A (e.g. Host institution does not have such facilities)

1. Does the summer student research program incorporate research rounds or teaching seminars as part of its curriculum?

Yes

No

1. If applicable: How often are research rounds or teaching seminars held for the students?

weekly

Biweekly

Monthly

1. How are the students required to present their work at the end of the term?

Internal Oral Presentation

External Oral Presentation

Internal Poster Presentation

External Poster Presentation

Manuscript submission for publications

None

1. Are students given awards for best oral and poster presentations?

Award for top oral presentations

Award for top poster presentations

None

Other:

**Section 7: Program Self-evaluation**

1. What proportion of students successfully publish their research projects in any type of format (ex. Peer-reviewed journal, non-peer reviewed journal, online; other formats, etc.)?

      %

Information not tracked centrally

Institution has record but does wish to disclose

1. Can eligible students who previously participated in the program return for another year?

Yes

No

1. If applicable: are students encouraged to participate in the program again (Ex. E-mails are sent out to former participants encouraging them to reapply)?

Yes

No

1. If applicable: How are students encouraged to participate in the program again?

Solely at the discretion of PI

Centrally (ex. E-mails are sent out to former participants encouraging them to reapply)

1. If applicable: Does the institution monitor retention rate (Number of eligible students that come back)?

Yes

No

1. Does the institution have a central process for collecting feedback?

Yes, student feedback is collected centrally

Yes, principal Investigator feedback is collected centrally

No central measures in place

1. If feedback is collected centrally, in what format is it collected and stored?

Written surveys

Electronic surveys

Not collected centrally

1. Does the program centrally track where students are in their training or career after completion of the program?

1-3 years later

3-5 years later

>5 years later

**Section 8: Other**

1. Does the program use social media to attract students?

Twitter

Facebook page

Other:

No use of social media

**Appendix 2:** Eligible Canadian summer student research programs (n = 91)

| **No.** | **Department or institution offering SSRP** | **Program name** |
| --- | --- | --- |
|  | **Ontario (n = 36)** | |
| 1 | Banting and Best Department of Medical Research | Charles Hollenberg Summer Studentship Program |
| 2 | Baycrest Centre | Baycrest Summer Student Program |
| 3 | Bloorview Research Institute | The Ward Family Summer Student Research Program |
| 4 | Institute for Clinical Evaluative Sciences (ICES) | CANHEART Summer Studentship Award |
| 5 | Samuel Lunenfeld Research Institute | RTC Summer Research Program for Undergraduates |
| 6 | North York General Hospital | Family Medicine Undergraduate Program |
| 7 | Keenan Research Centre | The Keenan Research Summer Student (KRSS) Program |
| 8 | Sunnybrook Research Institute | Hurvitz Brain Sciences Summer Student Research Program |
| 9 | Sunnybrook Research Institute | D+H SRI Summer Student Research Program |
| 10 | Sick Kids Research Institute | Starbucks Clinical Genetics/Genomics Research Studentship Award |
| 11 | Sick Kids Research Institute | The SickKids Summer Research (SSuRe) Program |
| 12 | University of Health Network (UHN) | Multi Organ Transplant Student Research Training Program (MOTSRTP) |
| 13 | University of Health Network (UHN) | ELLICSR Summer Research Undergraduate Program |
| 14 | Women's College Research Institute (WRCI) | WCRI Summer student research program |
| 15 | University of Toronto, Institute of Medical Sciences (IMS), | Summer Undergraduate Research Program (SURP) |
| 16 | Donnelly Centre | Donnelly Summer Undergraduate Student Program |
| 17 | University of Toronto, Biochemistry Department | Biochemistry Summer Student Research Project |
| 18 | University of Toronto, Institute of Biomaterial and Biomedical Engineering | IBBME Undergraduate Summer Research Program |
| 19 | University of Toronto, Immunology Department | Immunology Summer Research Program |
| 20 | University of Toronto, Laboratory Medicine & Pathobiology | LMP Summer Student Research Program |
| 21 | University of Toronto, Department of Medical Biophysics | Medical Biophysics Summer Student program |
| 22 | Ontario Genomics Institute | The Ontario Genomics Institute (OGI) Summer Research Fellowships in Genomics |
| 23 | University of Toronto, Department of Molecular Genetics. Research | Department of Molecular Genetics Undergraduate Summer Research Program |
| 24 | University of Toronto, MD Program | CREMS Summer Program |
| 25 | Children's Hospital of Eastern Ontario | CHEO Research Programs |
| 26 | The Ottawa Hospital | OHRI Summer Student Research Programs |
| 27 | The University of Ottawa Heart Institute | Ottawa Heart Institute Research Mentorship |
| 28 | University of Ottawa, Faculty of Medicine | Summer Studentship Program |
| 29 | University of Ottawa, Faculty of Medicine | 2016 Summer Studentship Program on Innovation in Undergraduate Medical Education |
| 30 | Queen's University, School of Medicine | School of Medicine Summer Studentships |
| 31 | Queen's University | Undergraduate Student Summer Research Fellowships (USSRF) |
| 32 | Lakehead University and Thunder Bay Regional Research Institute (TBRRI) | Summer School on Medical Imaging (SSMI) |
| 33 | Schulich Medicine & Dentistry | Summer Research Training Program (SRTP) |
| 34 | Schulich Medicine & Dentistry | Dean’s Undergraduate Research Opportunities Program 2016 (DUROP) |
| 35 | Schulich Medicine & Dentistry | Western University - Biochemistry Undergraduate Summer Research Program (BUSRP) |
| 36 | Schulich Medicine & Dentistry | Microbiology and Immunology Summer Undergraduate Research Fellowships (SURF) |
|  | **Alberta (n =16)** | |
| 37 | Alberta Cancer Foundation | Alberta Cancer Foundation 2016 Summer Studentship Awards |
| 38 | Women and children's health research institute | WCHRI Summer Studentships |
| 39 | Glenrose Rehabilitation Hospital | Glenrose Rehabilitation Hospital Summer Student Research Program |
| 40 | University of Alberta, Augustana campus | Augustana Summer Student Research Assistantships, Roger S. Smith Undergraduate Student Research Awards in partnership with Killam |
| 41 | University of Alberta, Faculty of Medicine and Dentistry | Undergraduate Summer Students' Research Program |
| 42 | University of Alberta, Faculty of Medicine & Dentistry | Family Medicine Summer Studentship |
| 43 | University of Alberta, Faculty of Medicine & Dentistry | EMeRG Summer Studentships |
| 44 | University of Alberta, Faculty of Medicine & Dentistry | 2016 Summer Studentship in Health Professions Education Grants Program |
| 45 | University of Alberta, Department of Medical Microbiology & Immunology | MMI Summer student program |
| 46 | University of Alberta, Faculty of Medicine & Dentistry | Department of Surgery Summer Studentship |
| 47 | University of Calgary | Program for Undergraduate Research Experience (PURE) Program |
| 48 | University of Calgary and University of Alberta | Health Solutions (AIHS) Summer Research Studentships |
| 49 | University of Calgary | Markin Undergraduate Student Research Program in Health and Wellness (USRP) |
| 50 | O'Brien Centre for the Bachelor of Health Sciences | O'Brien Centre Summer Studentships |
| 51 | Calgary Laboratory Services | CLS Undergraduate Summer Students |
| 52 | Calvin, Phoebe and Joan Snyder Institute for Chronic Diseases | Summer Studentships |
|  | **British Colombia (n =7)** | |
| 53 | The University of British Columbia, Faculty of Medicine | FoM Summer Student Research Program |
| 54 | The University of British Columbia, Faculty of Medicine | The Centre for Blood Research (CBR) Summer Studentship Program |
| 55 | The University of British Columbia, Faculty of Medicine | Faculty of Medicine (FoM) Department of Pathology & Laboratory Medicine Summer Student Fellowship |
| 56 | BC Children's Hospital Research Institute | Summer Student Research Program |
| 57 | BC Cancer Agency | 2016 BC Cancer Studentships |
| 58 | Centre for Heart Lung Innovation UBC and St. Paul's Hospital | Summer Student Research Program |
| 59 | The University of British Columbia, Faculty of Medicine | UBC Interstitial Lung Disease (ILD) Summer Studentship |
|  | **Quebec (n = 4)** | |
| 60 | McGill University, Faculty of Medicine Research and Graduate Studies Offices | Summer Research Bursary Program |
| 61 | McGill University, Faculty of Science | Science Undergraduate Research Awards (SURA) |
| 62 | McGill University, Institute for Health and Social Policy | Institute for Health and Social Policy (IHSP) Internship program |
| 63 | Meakins-Christie Laboratories | Meakins-Christie Studentships |
|  | **New Brunswick (n = 1)** | |
| 64 | New Brunswick Health Research Foundation | NBHRF Summer Studentship Program |
|  | **Nova Scotia (n = 5)** | |
| 65 | IWK Health Centre | IWK Undergraduate Student Summer Research Program |
| 66 | Dalhousie University, Faculty of Medicine | RIM - Research in medicine |
| 67 | Dalhousie University, Faculty of Science | Summer Research Program for Non-Medical Students |
| 68 | Dalhousie University, Faculty of Medicine | Summer Student Research Program (Medical Students) |
| 69 | Dalhousie University, Faculty of Medicine | Summer Student Research Program (Undergraduate Students) |
|  | **Newfoundland (n = 3)** | |
| 70 | Memorial University, Faculty of Medicine | Summer Undergraduate Research Award (SURA) program - medical students only |
| 71 | Memorial University, Faculty of Medicine | Summer Undergraduate Research Award (SURA) program - medical students and non-medical students |
| 72 | Memorial University | University Student Summer Internship Program (USSIP) |
|  | **Saskatchewan (n = 2)** | |
| 73 | Saskatchewan College of Medicine | Dean's summer research projects |
| 74 | Saskatchewan College of Medicine | Biomedical Summer Research Projects |
|  | **Manitoba (n = 4)** | |
| 75 | Children Hospital Research Institute of Manitoba | CHRIM Undergraduate Summer Studentships |
| 76 | Diagnostic Services Manitoba | DSM Summer Studentship Program |
| 77 | University of Manitoba, Faculty of Medicine | BScMed Summer Research Program |
| 78 | University of Manitoba, Faculty of Medicine | MedII Summer Student Research Program |
|  | **Pan-Canadian (n = 13)** | |
| 79 | Canadian Association of Gastroenterology | Summer Studentship Awards |
| 80 | Canadian Blood Services | Canadian Blood Services Summer Research Scholarship Program |
| 81 | Canadian Cystic Fibrosis Canada | Studentship Awards |
| 82 | Canadian Liver Foundation | Canadian Liver Foundation Summer Studentships |
| 83 | Canadian Hemophilia Society | Summer Studentships in Inherited Bleeding Disorders Research |
| 84 | Canadian Rheumatology Association | CRA Summer Studentship |
| 85 | Eye Foundation of Canada | Student Scholarship Program (Summer) |
| 86 | Canadian Society for the History of Medicine | Hannah Studentship |
| 87 | Canadian Frailty Network | CFN Summer Student Awards |
| 88 | Mach-Gaensslen Foundation of Canada | Summer Student Research Program |
| 89 | Canadian Foundation for Infection Disease | AMMI Canada Medical Student Research Award |
| 90 | Brain Tumour Foundation of Canada | The Brain Tumour Research Studentship Program |
| 91 | The Canadian Society for Vascular Surgery | 2016 National Student Research Award |

**Appendix 3:** Comparison of institutional types offering summer student research programs across Canada in 2016

| **Provinces** | **Institutional types** | | | | **Total** |
| --- | --- | --- | --- | --- | --- |
|  | **Faculty of Medicine** | **Hospital and hospital associated research institute** | **Research Institute** | **University** |  |
| **Ontario** | 8 | 14 | 5 | 9 | 36 |
| **Alberta** | 4 | 1 | 3 | 8 | 16 |
| **British Colombia** | 4 | 2 | 1 | 0 | 7 |
| **Quebec** | 1 | 0 | 1 | 2 | 4 |
| **New Brunswick** | 0 | 0 | 1 | 0 | 1 |
| **Nova Scotia** | 3 | 1 | 0 | 1 | 6 |
| **Newfoundland** | 2 | 0 | 0 | 1 | 3 |
| **Saskatchewan** | 2 | 0 | 0 | 0 | 2 |
| **Manitoba** | 2 | 1 | 1 | 0 | 4 |
| **Total** | 26 | 19 | 12 | 21 | 13 |

**Appendix 4:** Disciplines of research offered by Canadian SSRPs (n = 46) for 2016. Choices were not mutually exclusive.


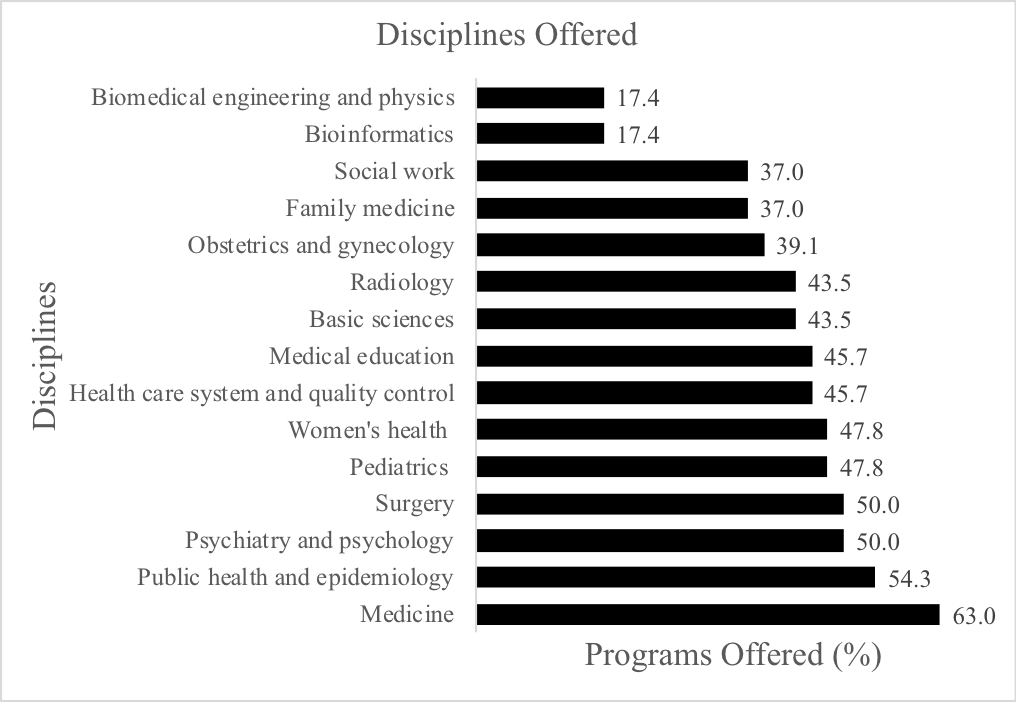


**Appendix 5:** Logic model providing a framework of core outcome sets to guide program evaluation of SSRPs

| **Inputs**  Resources dedicated to or consumed by program | **Activities**  How the program uses the inputs to achieve its goals | **Outputs**  Direct outputs as a result of program activities | **Program-Related Outcomes (Proximal Outcomes)**  Short, intermediate and long-term benefits of program activities |
| --- | --- | --- | --- |
| - Funding to support summer studentship - Faculty time - Program coordinator and/or central oversight committee - Research facility to host student research projects - Leadership support - Curricular design expertise | ***For programs:***   - - Advertise program, screen/select applicants and research supervisors   - Program administration and logistics   - Program evaluation   ***For Students:***   - - Professional networking opportunities   - Clinical observership opportunities   - Educational and research training sessions and/or supplementary online educational curriculum (curriculum design, design and evaluation)   - Summer student social events   ***For Supervisor:***   - - Workshops and training sessions on mentoring   - Networking opportunities | ***For programs:***   - - Number of applicants and participants   - Number of completed research projects   - Number of “hits” on program website   ***For Students:***   - - Number of student publications   - Number of student presentations and published abstracts   - Number of protected one-on-one time with supervisor   - Number of research awards   - Participation level in educational/teaching rounds   - Number of completed online supplementary educational modules   ***For Supervisor:***   - - Number of mentored research students   - Amount of time spent training and mentoring students   - Number of educational and teaching awards   - Number of student publications   - Number of student presentations and published abstract | ***For Students:***   - - Improved research knowledge and skills^†^   - Enhanced self-efficacy and identity as a researcher^†^   - Resume building for future jobs/professional school applications/residency^†^   - Managing commonly faced realities of research (e.g. tight research deadlines, limited resources, etc.) ^†^   - Improve writing and oral presentation ability^†^   - Improved knowledge of topics presented within curriculum^†^   - Strengthen professional network and support systems^†^   - Increase confidence and interest in conducting research^†^   - Earlier identification of medicine/surgical specialty interests (e.g., geriatrics, rural, radiology) ^†^   - Increased awareness of research application^†^   - Evidence of continued commitment to developing a research career^‡^   - Subsequent research initiated that includes networks developed during SSRP^‡^   - Experiential learning: broadening/reinforcing learning through completion of a subsequent research project^‡^   - Improve role transition into clinician-scientist^§^   - Foster and strengthen interest to pursue postgraduate research training/degree(s) ^§^   - Community of practice that supports research career development ^§^   - Community of SSRP graduates who promote research by giving back to their program ^¥^   ***For Supervisors:***   - - Networking for mentors^†^   - Strengthening mentorship skills (e.g., providing feedback, setting expectations) ^†^   - Research team management skills^‡^   - Subsequent graduate student recruitment/supervision of SSRP students^‡§^   ***For Program:***   - - Demonstrating recognition and value of research^‡^   - Forming a community of researchers (faculty and trainees) ^‡^   - Refinement of goal setting and evaluative strategies^‡^   - Developing future research leaders^§^ |
|  |  |  | **System-Wide Outcomes (Distal Outcomes)** |
|  |  |  | - - Increase number of clinician-scientists^§^   - Increase researcher diversity^§^   - Increase knowledge translation^§^   - Program graduates who are editors of journals^§^ |
| **Assumptions** | - Both positive and negative mentorship significantly impacts the decision to pursue research and medical speciality | | |
| **External Factors** | - Graduate programs are highly competitive - Length of summer student research programs may make some activities difficult to complete | | |

*SSRP: Summer student research program; ^†^Short (≤1 year), ^‡^intermediate (1-3 year) and ^§^long-term (≥3 years) outcomes provided
